# Development of hypobranchial muscles with special reference to the evolution of the vertebrate neck

**DOI:** 10.1186/s40851-018-0087-x

**Published:** 2018-02-18

**Authors:** Noritaka Adachi, Juan Pascual-Anaya, Tamami Hirai, Shinnosuke Higuchi, Shigeru Kuratani

**Affiliations:** 1 Evolutionary Morphology Laboratory, RIKEN center for Developmental Biology, 2-2-3 Minatojima-minami, Chuo-ku, Kobe, 650-0047 Japan; 2 Department of Biology, Graduate School of Science, Kobe University, Kobe, 657-8501 Japan

**Keywords:** Head–trunk interface, Hypobranchial muscles, Pericardium, Pharyngeal arch

## Abstract

**Background:**

The extant vertebrates include cyclostomes (lamprey and hagfish) and crown gnathostomes (jawed vertebrates), but there are various anatomical disparities between these two groups. Conspicuous in the gnathostomes is the neck, which occupies the interfacial domain between the head and trunk, including the occipital part of the cranium, the shoulder girdle, and the cucullaris and hypobranchial muscles (HBMs). Of these, HBMs originate from occipital somites to form the ventral pharyngeal and neck musculature in gnathostomes. Cyclostomes also have HBMs on the ventral pharynx, but lack the other neck elements, including the occipital region, the pectoral girdle, and cucullaris muscles. These anatomical differences raise questions about the evolution of the neck in vertebrates.

**Results:**

In this study, we observed developing HBMs as a basis for comparison between the two groups and show that the arrangement of the head–trunk interface in gnathostomes is distinct from that of lampreys. Our comparative analyses reveal that, although HBM precursors initially pass through the lateral side of the pericardium in both groups, the relative positions of the pericardium withrespect to the pharyngeal arches differ between the two, resulting in diverse trajectories of HBMs in gnathostomes and lampreys.

**Conclusions:**

We suggest that a heterotopic rearrangement of early embryonic components, including the pericardium and pharyngeal arches, may have played a fundamental role in establishing the gnathostome HBMs, which would also have served as the basis for neck formation in the jawed vertebrate lineage.

**Electronic supplementary material:**

The online version of this article (10.1186/s40851-018-0087-x) contains supplementary material, which is available to authorized users.

## Background

**Fig. 1:**
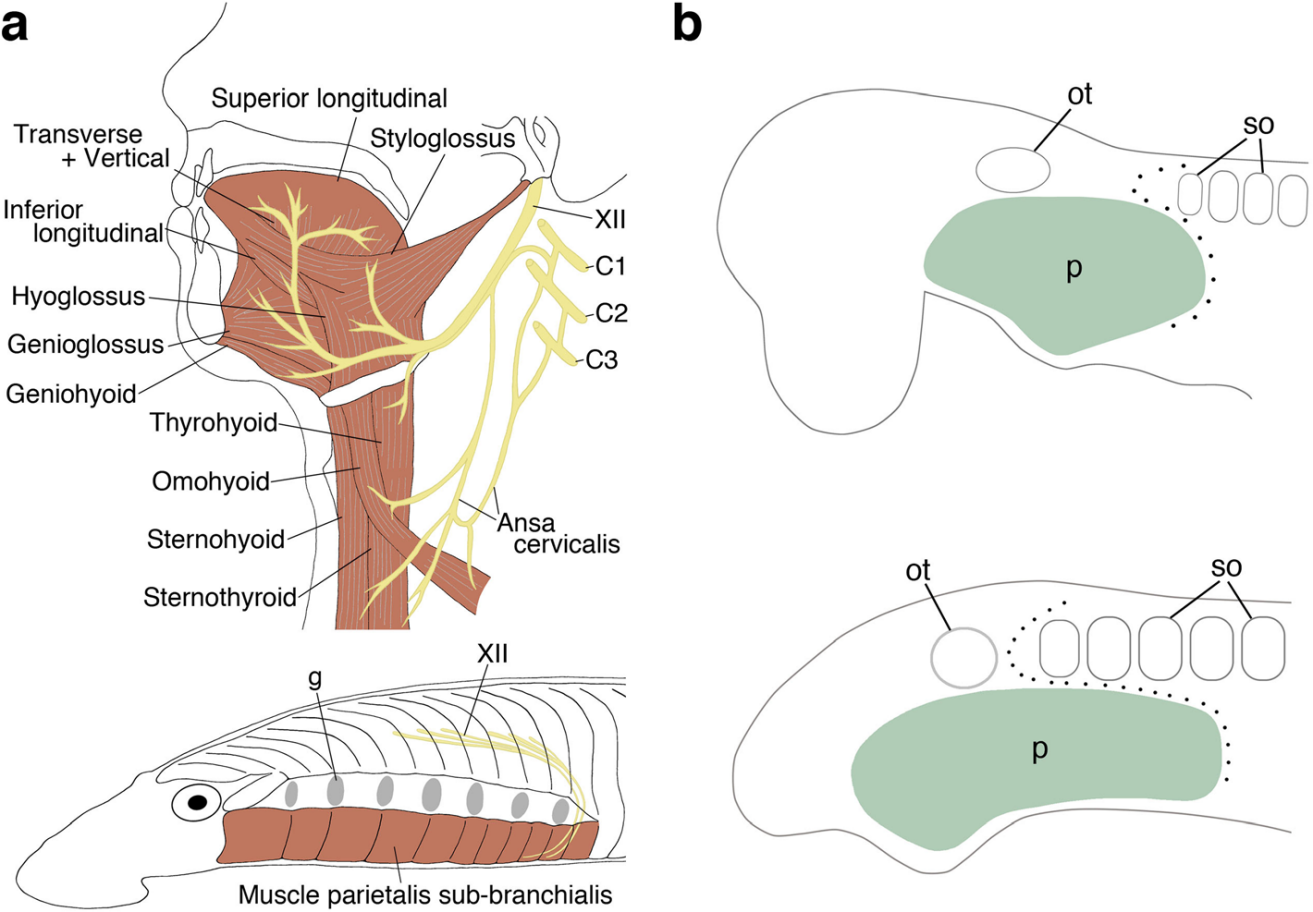
Vertebrate HBMs and the head–trunk interface. Human HBMs are composed of multiple voluntary muscles innervated by the hypoglossal nerve (XII) and cervical spinal nerves (C1–3) (**a**, top). The lamprey also possesses HBMs and the hypoglossal nerve (**a**, bottom). Schematic diagram of the head–trunk interface in gnathostomes (**b**, top) and the lamprey (**b**, bottom). Dotted lines indicate the interface. C1–3, cervical spinal nerves; g, gill opening; ot, otic vesicle; p, pharyngeal arches; so, somites

Based on diverse anatomical features, such as the jaw, nostril, and paired appendages, all living vertebrates are categorized into two major groups, cyclostomes and gnathostomes. The neck is one of the gnathostome features, found caudal to the interface of the head and trunk [1], defined as a domain between the occipital part of the cranium and the shoulder girdle of the gnathostomes, and occupied by cucullaris and hypobranchial muscles (HBMs) [2–6]. The HBMs are positioned in the ventral aspect of the neck and pharynx, and are collectively innervated by the hypoglossal and/or the occipitospinal nerves. These muscles function in jaw opening, swallowing, and respiration in the gnathostomes. In tetrapods, the rostral part of the muscles constitutes the tongue and the posterior part forms the infrahyoid musculatures, performing diverse functions from feeding to speech (Fig. 1a) [7–15].

Morphologically, cyclostomes do not have an evident neck domain, as the occipital region, shoulder girdle, and cucullaris muscles are not present in these animals [3, 16]. In contrast, both lampreys and hagfishes have HBMs on the ventral part of the pharynx; these are innervated by the homologue of the hypoglossal nerve (Fig. 1a) [3, 17]. However, these muscles lie superficial to the pharynx, not inside the mouth and pharynx as they do in the gnathostomes [18, 19]. These anatomical disparities raise questions about the evolutionary origins of the neck in gnathostomes; however, the mechanisms leading to these disparities remain largely unknown.

The anatomical elements constituting the neck originate in the vicinity of the head–trunk interface of gnathostome embryos. The interface consists of the anterior extremity of somites and the posterior extremity of pharyngeal arches, and the circumpharyngeal crest cells pass through this interfacial domain (Fig. 1b) [1, 20]. Notably, the anterior occipital somites and circumpharyngeal crest cells give rise to HBMs and their connective tissues, respectively [4, 21, 22] also see below); thus, the interfacial domain is a key embryonic region for understanding neck morphogenesis [1].

As noted, although anatomical disparities exist between cyclostomes and gnathostomes, a number of embryological studies have observed resemblances in the head–trunk interface and HBMs. For example, it has been shown that the lamprey embryo also possesses the interfacial domain [16, 23]. Studies have revealed that the occipital somites give rise to HBM precursors, which migrate once posterior to the pharyngeal arches and then turn rostrally to reach the ventral pharynx in vertebrates, including the lamprey [4, 9, 22, 24–33]. Molecular analyses have revealed the involvement of *Pax3*, a paired box transcription factor-encoding gene, in the development of HBMs [26, 28, 29, 34]. Similarly, lamprey HBMs are marked by *Pax3/7*, a homolog of *Pax3* [35]. Thus, cyclostomes and gnathostomes have similar embryonic components and diverse anatomical outcomes in the head–trunk interface. This suggests that developmental differences of components in the interfacial domain account for the anatomical disparities between the two groups, and comparative embryological analysis of this domain is crucial to understanding vertebrate neck evolution [3]. The analysis of HBMs is especially crucial, as muscles found in both groups would be a useful developmental landmark for comparing the interfacial domains in these animals. However, no detailed comparison of the HBM pathway between lamprey and gnathostome embryos has been reported to date. Furthermore, the migration trajectory of HBMs relative to the pharynx remains obscure, even in embryos of gnathostomes. In the present study, we investigated the development of HBMs and the embryonic morphology of the head–trunk interface in chicken, mouse, shark, and lamprey embryos to gain insight into the evolution of the vertebrate neck.

## Methods

### Sample collection

Mature adult lampreys (*Lethenteron camtschaticum,* synonym for *Lethenteron japonicum*) were collected in Hokkaido, Japan, and the fertilized eggs were obtained as described previously [36]. Adult cloudy catsharks (*Scyliorhinus torazame*) were captured in Ibaraki, Japan. They laid fertilized eggs in saltwater tanks and the eggs were incubated at 16 °C. Fertilized chicken eggs (*Gallus gallus*) were purchased from local suppliers and incubated at 38 °C. C57BL/6 mouse embryos (*Mus musculus*) were obtained from CDB animal facilities. Wnt1-Cre heterozygous mice [37] and R26R-H2B-EGFP homozygous mice (Acc No. CDB0203K: http://www2.clst.riken.jp/arg/reporter_mice.html) [38] were crossed and the offspring were genotyped by PCR to obtain Wnt1-Cre/R26R-H2B-EGFP embryos. Noon on the day that the vaginal plug was observed was defined as E0.5. Embryos of lamprey, shark, chicken, and mouse were staged, following the systems described in [39–42], respectively.

### Histological analysis and 3D reconstruction

Lamprey and shark embryos were fixed in Bouin’s fixative, sectioned, and stained as described previously [43]. At least three embryos were observed for each developmental stage. Carazzi’s hematoxylin was used for lamprey sections, Mayer’s hematoxylin for shark sections, and Alcian blue for cartilaginous staining. 3D reconstruction of embryos was performed by the method described previously [43].

### Molecular cloning and phylogenetic analysis

Embryonic total RNA was extracted by TRIzol Reagent (Life Technologies), and cDNA was synthesized by SuperScript IV Reverse Transcriptase (Thermo Fisher Scientific). RNA-sequence analysis of embryonic lamprey and shark was performed and the sequences of lamprey *Nkx2–3/2–5/2–6*, *Tbx1/10A*, and *Tbx4/5*, and shark *Nkx2–5* and *Tbx5* were found in the assembled data set (to be published elsewhere). DNA fragments were amplified, cloned, and sequenced as described previously [44]. The accession numbers and primers used in this study are listed in the Additional file 1. The sequences of lamprey *DlxB*, *Hox2α*, *Hox3α*, and *Pax3/7*, and shark *Dlx5*, *Pax3*, and *Tbx1* have been described previously [35, 44–48]. Shark *Hoxa2* sequence has been described recently in a separate study (Pascual-Anaya et al., under preparation) and is publicly available in GenBank under the accession number MF398238. For phylogenetic analyses, amino acid sequences of orthologous genes’ from different vertebrate species were compiled from GenBank (http://www.ncbi.nlm.nih.gov/) and Ensembl (http://www.ensembl.org/index.html). Multiple alignment of protein sequences were performed with MAFFT [49] as implemented at the European Bioinformatics Institute’s web server (http://www.ebi.ac.uk/Tools/msa/mafft/), or MUSCLE [50] as implemented in MEGA v7 [51], release 7,161,111-i38651, with default parameters, and saved in FASTA format. Resulting alignments were then trimmed by trimAl version 1.2rev59 [52] using the ‘–automated1’ parameter and then formatted into NEXUS format by readAl v.1.2 (bundled with the trimAl package) (Suppl. Molecular phylogenetic trees of lamprey were inferred using Bayesian inference with MrBayes 3.2.6 [53] under the assumption of an LG + I + G evolutionary model and were performed with two independent MrBayes runs, 4 chains each. Convergence was considered when the standard deviation < 0.01 for more than 1 million generations. To build the consensus tree, a burn-in of the 25% of the trees was performed (see Additional file 1: Figure S1 for the number of generations of each gene’s phylogenetic analysis).

### In situ hybridization

Lamprey and shark embryos were fixed in MEMFA fixative. Chicken and mouse embryos were fixed in 4% PFA/PBS. Whole-mount in situ hybridization was performed as described in [54]. The method of two-color whole-mount in situ hybridization has been described by [55]. Embryos were imaged using a Leica MZ16FA. After whole-mount in situ hybridization, lamprey embryos were immersed in MEMFA fixative with 2.5% glutaraldehyde solution, dehydrated, and embedded in paraffin. Sections were prepared at 6 μm, and counterstained in eosin solution. The protocol of in situ hybridization on shark sections was described in [36]. Adjacent paraffin sections were used to compare gene expression patterns. Sections were imaged with an Olympus BX53. The expression pattern of each gene was investigated in > 3 embryos.

### Immunohistochemistry

Three Wnt1-Cre/R26R-H2B-EGFP embryos were obtained and utilized in the analysis. The embryos were fixed in 4%PFA/PBS, dehydrated, and embedded in paraffin. Sections were prepared at 6 μm. Deparaffinized sections were autoclaved in 1 mM citrate buffer (pH 6.0) to unmask epitopes [56]. Anti-PAX3 mouse monoclonal antibody (1/100 dilution, ab69856, Abcam) and anti-GFP rabbit polyclonal antibody (1/500 dilution, ab290, Abcam) were used for the primary antibody. Goat anti-mouse IgG Alexa Fluor 647 (1/200 dilution, A21237, Thermo Fisher Scientific) and goat anti-rabbit IgG Alexa Fluor 488 (1/500 dilution, A11034, Thermo Fisher Scientific) were used for the secondary antibody. The signal detection was performed with a confocal laser microscope Leica TCS SP8X and Spectral Dye Separation in LAS AF software.

## Results

### Development of HBMs in jawed vertebrate embryos

**Fig. 2:**
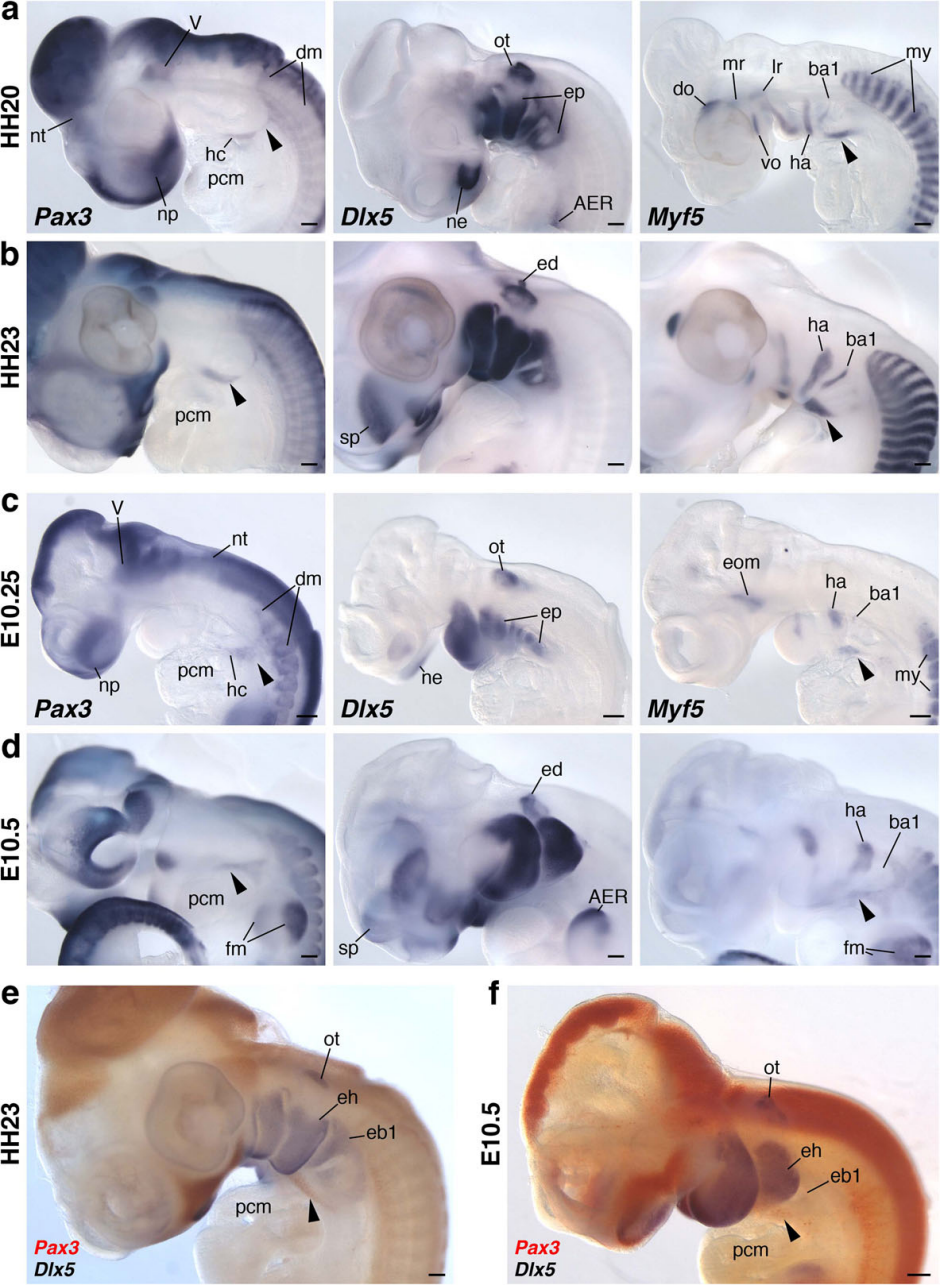
Gene expression analysis of HBM precursors in chicken and mouse embryos. *Pax3*, *Dlx5* and *Myf5* expression patterns in chicken HH20 (**a**) and HH23 (**b**), and mouse E10.25 (**c**) and E10.5 (**d**). Two-color in situ hybridization with *Pax3* (red) and *Dlx5* (blue) probes in chicken HH23 (**e**) and mouse E10.5 (**f**). Lateral views. Arrowheads indicate HBM precursors. Note that HBM precursors, marked by *Pax3* and *Myf5* expressions, developed medially at the level of hyoid arch at stage HH23 and E10.5 embryos. AER, apical ectodermal ridge; ba1, first branchial arch mesoderm; dm, dermomyotome; do, dorsal oblique; eb1, ectomesenchyme of first branchial arch; ed., endolymphatic duct; eh, ectomesenchyme of hyoid arch; ep, ectomesenchyme of pharyngeal arches; fm, forelimb muscles; ha, hyoid arch mesoderm; hc, hypoglossal cord; lr, lateral rectus; mr, medial rectus; my, myotome; ne, nasal epithelium; np, nasal prominence; nt, neural tube; ot, otic vesicle; pcm, pericardial mesoderm; sp., subpallium; V, trigeminal ganglion; vo, ventral oblique. Scale bars, 200 μm

We first examined the development of HBMs in chicken and mouse. To this end, we observed *Pax3* and *Myf5* expressions to monitor the embryonic HBMs [29, 34] and *Dlx5* expression as the marker for the ectomesenchyme of pharyngeal arches [57]. In both species (HH20 chicken and E10.25 mouse embryos), the HBM precursors, labeled by *Pax3* riboprobes, were extended from the ventral part of the occipital somites and continued ventrally as a bundle of *Pax3*-positive cells, making the so-called hypoglossal cord [26–29]. *Myf5* expression was only detected in the anteroventral compartment of the cord. The hypoglossal cord circumvented the posterior boundary of the pharynx with *Dlx5*-positive mesenchyme, and the rostral part of the cord reached to the ventrocaudal aspect of the pharyngeal arches (Fig. 2a, c).

Subsequently, these precursors reached to the lateral side of the anterior pericardium at HH23 and E10.5 (Fig. 2b, d–f). In these embryos, the pericardium was commonly located ventral to the pharynx, and the rostral tip of HBMs appeared to have penetrated the medial side of the hyoid arch (Fig. 2e, f). This medial extension of HBM precursors was also suggested by the expression pattern of *Myf5*, which marks both cranial and trunk myoblasts (Fig. 2a–d).

**Fig. 3:**
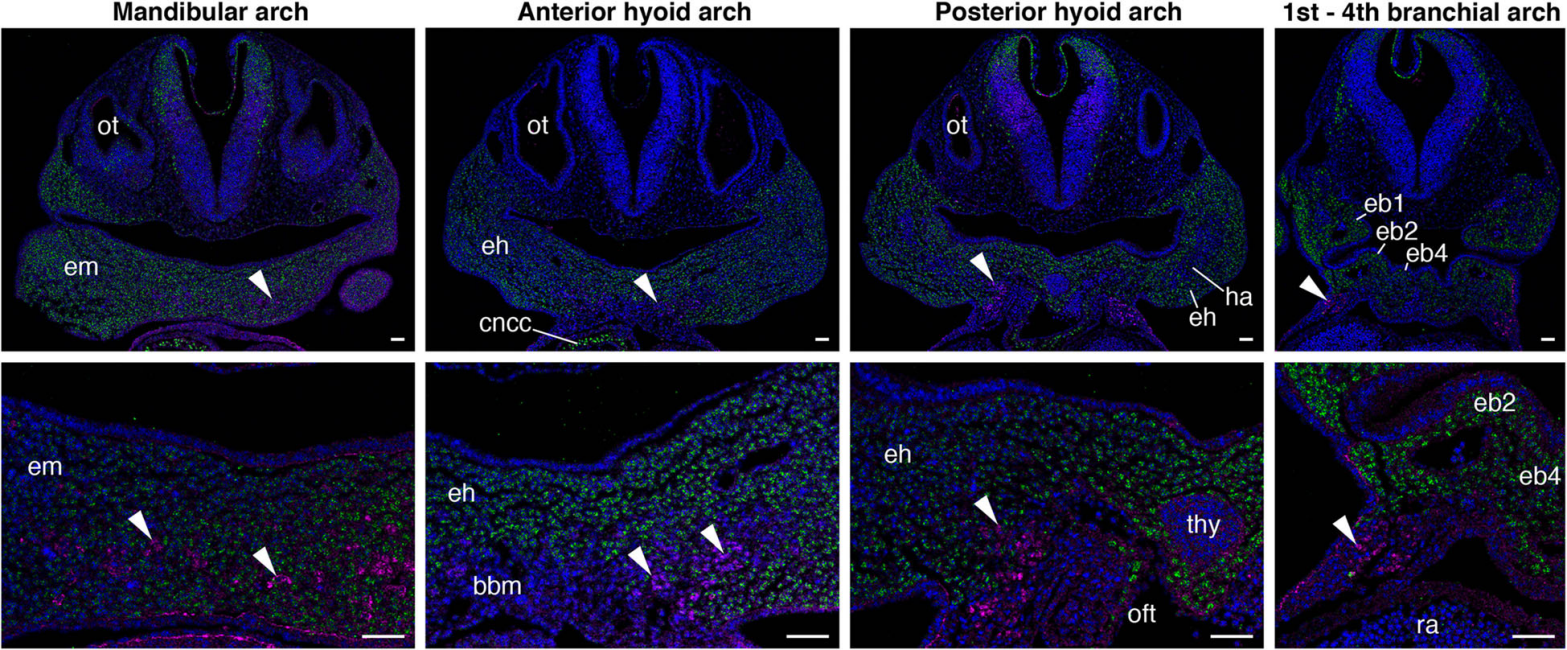
Trajectory of HBM precursors in mouse embryos. Transverse sections of mandibular, hyoid and branchial arch levels in Wnt1-Cre/R26R-H2B-EGFP embryos at stage E10.5. Bottom panels show the magnification of pharyngeal floor. Arrowheads indicate HBM precursors. PAX3 protein was detected on both sides of the EGFP-negative basibranchial mesenchyme and laterally surrounded by EGFP-positive neural crest cells at the hyoid arch. Bbm, basibranchial mesenchyme; cncc, cardiac neural crest cells; eb1–4, ectomesenchyme of first to fourth branchial arch; eh, ectomesenchyme of hyoid arch; em, ectomesenchyme of mandibular arch; ha, hyoid arch mesoderm; oft, outflow tract; ot, otic vesicle; ra, right atrium; thy thyroid gland. Scale bars, 50 μm

To visualize the distribution of HBM precursors and pharyngeal arches more precisely, we performed immunohistochemistry of PAX3 on Wnt1-Cre/R26R-H2B-EGFP mouse embryos, which rigorously mark the ectomesenchyme of pharyngeal arches with EGFP (Fig. 3 and Additional file 1: Figure S2). At the level of postotic pharyngeal arches (third to sixth arches), the HBM precursors, immunostained with PAX3 antibody, were detected lateral to the EGFP-positive ectomesenchyme and within the pericardial mesoderm (Fig. 3). This indicates that the HBMs are not covered by the posteriormost ectomesenchyme while traveling the posterior edge of the pharynx. The PAX3-positive cells extended from the pericardiac region to the ventromedial portion of the hyoid arch, and lay on both sides of the basibranchial mesenchyme, which is not marked by EGFP antibody. The precursors were laterally enclosed by EGFP-positive ectomesenchyme at the hyoid arch level, and completely surrounded by the ectomesenchyme at the floor of the mandibular arch (Fig. 3). The HBM precursors thus passed through the posterior edge of the caudal pharynx, along the lateral side of the pericardium, ventral to branchial arches, and then inside the mandibular and hyoid arches in chicken and mouse embryos.

We next explored the developmental pathway of HBMs in the cloudy catshark, *S. torazame*, to test whether the trajectory of HBM precursors is shared in crown gnathostomes. We performed 3D reconstruction of shark embryos from the otic vesicle to heart levels (Additional file 1: Figures S3a and S4). Shark mesoderm, which exhibits an epithelialized, not mesenchymal, state at early stages [43, 58], is a useful model for observing HBMs in gnathostomes.

**Fig. 4:**
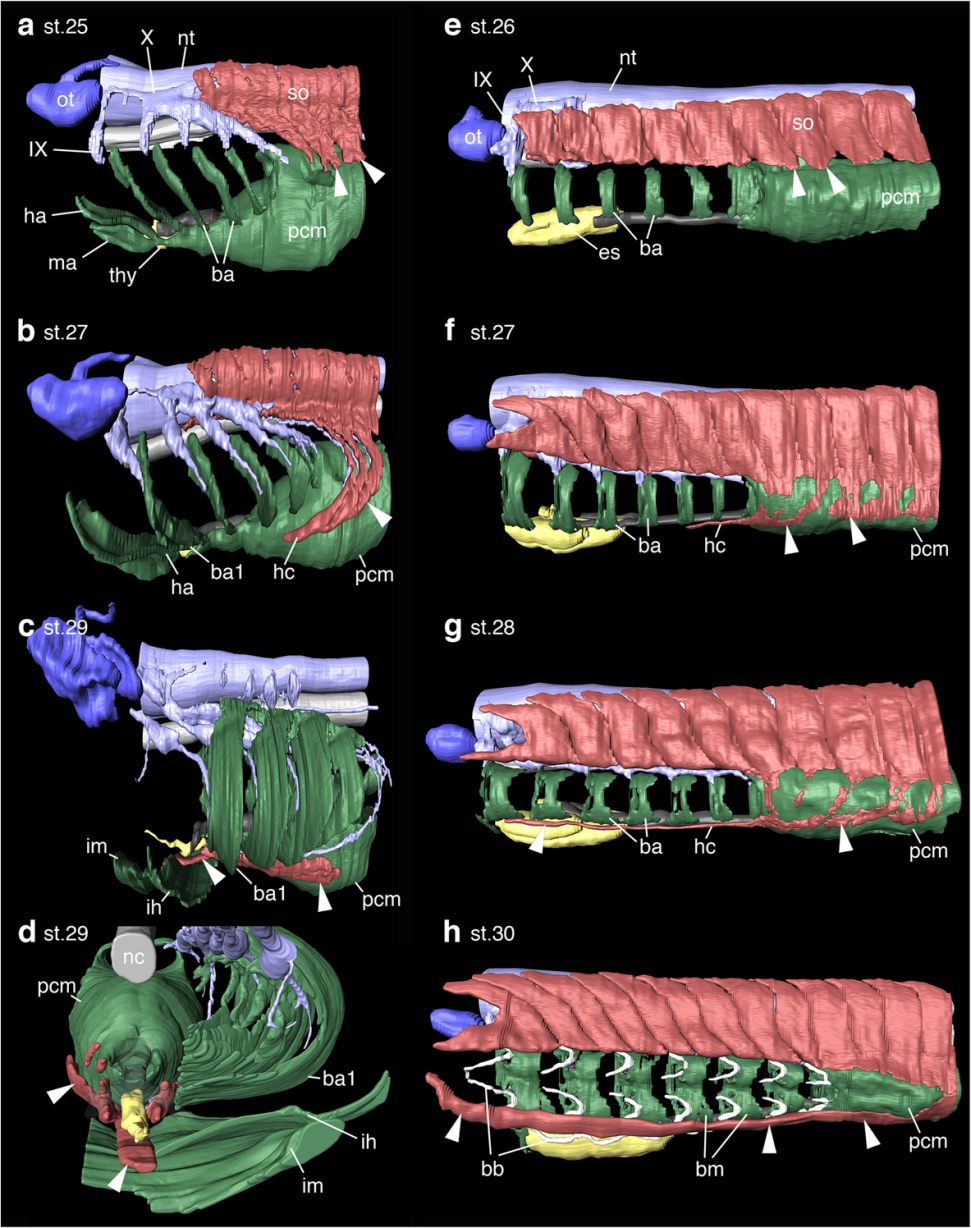
Developmental sequence of HBMs. **a**-**h**, 3D reconstruction images of shark (**a**-**d**) and lamprey (**e**-**g**) embryos from lateral view. (**d**) Anterior view of (**c**) with translucent ventral aorta. Somites developed ventrally sending HBM precursors (arrowheads) lateral to the pericardial mesoderm in both species (**a**, **b**, **e** and **f**). HBM precursors developed inward being surrounded laterally and ventrally by mandibular and hyoid muscles in sharks (**c**, **d**), whereas the precursors extended outside the branchial muscles and skeletons in lampreys (**g**, **h**). Somites detached from HBM precursors (**c**) and right pharyngeal muscles (**d**) are not shown. ba, branchial arch mesoderm; ba1, first branchial arch mesoderm; bb, branchial basket; bm, branchial muscles; es, endostyle; ha, hyoid arch mesoderm; hc, hypoglossal cord; ih interhyoideus; im, intermandibularis; ma, mandibular arch mesoderm; nc, notochord; nt, neural tube; ot, otic vesicle; pcm, pericardial mesoderm; so, somites; thy thyroid gland; IX, glossopharyngeal nerve; X, vagus nerve

**Fig. 5:**
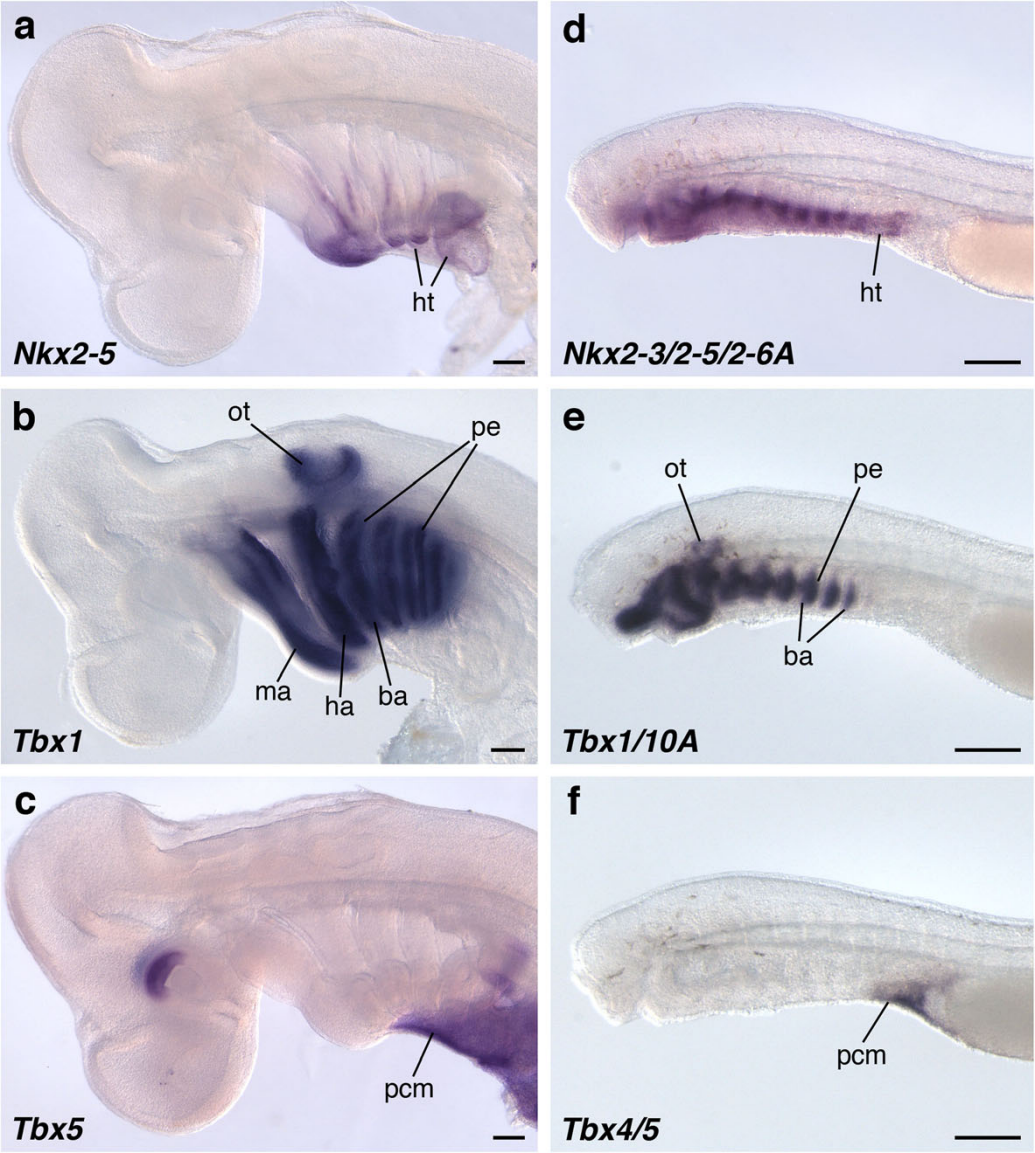
The relative position of the pericardium and pharyngeal arches. *Nkx2–5* (**a**), *Tbx1* (**b**), and *Tbx5* (**c**) expressions in *S*. *torazame* at the stage 25 from the lateral view. *Nkx2–3/2–5/2-6A* (**d**), *Tbx1/10A* (**e**), and *Tbx4/5* (**f**) expressions in the lamprey at the stage 26 from the lateral view. *Nkx2–5* and *Tbx5* homologs were expressed in the cardiac mesoderm. *Tbx1* cognate genes were detected in the pharyngeal arch mesoderm and endoderm. Note that the cardiac mesoderm resided ventral to pharyngeal arches in shark embryos, whereas the cardiac mesoderm lay posterior to the arches in lamprey embryos. ba, branchial arch mesoderm; ha, hyoid arch mesoderm; ht., heart tube; ma, mandibular arch mesoderm; ot, otic vesicle; pcm, pericardial mesoderm; pe, pharyngeal endoderm. Scale bars, 200 μm

At stage 25, *S. torazame* embryos showed mesoderm of seven pharyngeal arches located dorsal to the pericardium (Figs. 4a, 5a-c, and Additional file 1: Figure S4a, e). This relative position of the pericardium ventral to the pharynx was also observed in the early stages of the embryos, resembling that of chicken and mouse embryos (Fig. 2 and Additional file 1: Figure S5). The rostral somites formed ventral protrusions, forming a posteriorly oriented arc behind the pharynx, which extended toward the pericardium, showing the initial migration of the HBMs, as described previously (Fig. 4a) [40].

By stage 27, HBM precursors had descended around the posterior edge of the branchial arches to form the hypoglossal cord [27] on each side of the pericardium. The rostral tip of the cord had reached to the level of the fourth branchial arch (Fig. 4b).

At stage 29, the HBM precursors extended medially at the level of the hyoid arch. Notably, its anterior part was embraced laterally and ventrally by the intermandibularis and interhyoideus muscles, while the posterior remained ventral to the branchial arches (Fig. 4c, d). Crucially, this topographical relationship of HBMs and pharyngeal arches in this species perfectly coincided with that of chicken and mouse (Figs. 2, 3 and 4). The HBM precursors branched out to form the coracohyoideus and coracobranchialis muscles on both sides of the ventral aorta and pericardium, and subsequently, the majority of the HBMs were found inside gill muscles and cartilages (interhyoideus and constrictor superficialis muscles, ceratohyal, epibranchial, and ceratobranchial cartilages), as seen in the adult shark (Fig. 4c, d, and Additional file 1: Figure S4).

### Development of HBMs in the lamprey

To compare the developmental trajectory of HBMs between cyclostomes and gnathostomes, we observed the development of the Arctic lamprey, *L. camstchaticum* (Additional file 1: Figures S3b and S6). The youngest lamprey embryos examined in histological analysis were at Tahara’s stage 26 [39], when yolk granules were substantially reduced. Each of the pharyngeal arches possessed branchial mesoderm, which was located anterior to the pericardial mesoderm, immediately below the axial mesoderm (Figs. 4e, 5d–f, and Additional file 1: Figure S6a, e). This topology of the pharyngeal mesoderm and pericardium was also observed in earlier stages of lamprey embryos (Additional file 1: Figure S7). Thus, the relative position of the pericardium posterior to the pharynx contrasts with that in gnathostomes (Additional file 1: Figures S5 and S7). Signs of HBM development in the lamprey were first observed on the ventrolateral edge of somites, above the pericardial mesoderm (Fig. 4e and Additional file 1: Figure S6a, e, j-k').

During stages 27 and 28, the HBM precursors on both sides extended ventrally between the ectoderm and the pericardial mesoderm as several streams of cell populations (Fig. 4f and Additional file 1: Figure S6). These streams later converged ventroanteriorly to form the hypoglossal cord [27], which expands from the lateral side of the pericardium to the posterior branchial arches (Fig. 4f, g). Although the trajectory of these HBMs spreading on the lateral side of the pericardium is similar to that in gnathostomes, the lamprey HBMs uniquely developed along the ventrolateral aspect of the branchial arches (no longer in the pericardial mesoderm as in gnathostomes), differing clearly from those in gnathostomes (Figs. 2, 3 and 4).

By stage 30, HBM precursors developed further anteriorly up to the otic level and resided ventrolaterally to the pharyngeal muscles and skeletons, including mandibular and hyoid arches (Fig. 4g and Additional file 1: Figure S6). This relative position of HBMs and the pharynx is comparable with that in the adult lamprey.

### Trajectory of the HBMs relative to pharyngeal arches

To show the distribution of HBMs and pharyngeal arches in shark and lamprey embryos more clearly, we performed in situ hybridization using orthologous genes of *Pax3* and *Dlx5*, as well as *Tbx1*, which is expressed in the head mesoderm of pharyngeal arches, and *Hox* genes, which mark ectomesenchymal cells in the pharyngeal arches (Additional file 1: Figure S1) [35, 44, 45, 47, 59].

**Fig. 6:**
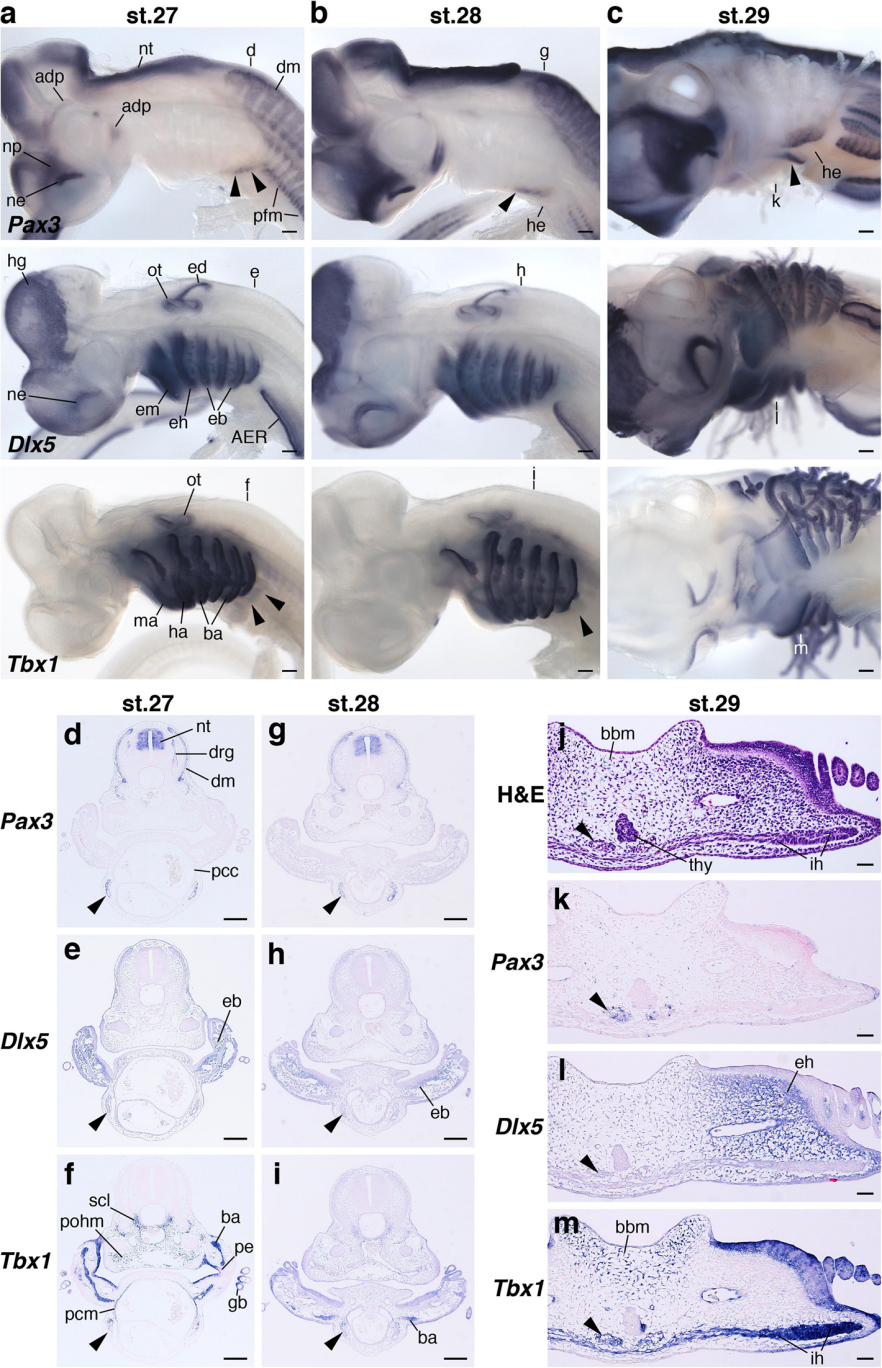
The arrangement of HBMs pathways and pharyngeal arches in shark embryos. *Pax3*, *Dlx5* and *Tbx1* expressions in sharks at stage 27 (**a**), 28 (**b**) and 29 (**c**). (**a**, **b**) Lateral and (**c**) ventrolateral views. Section in situ hybridization of *Pax3*, *Dlx5* and *Tbx1* at stage 27 (**d**–**f**), 28 (**g**–**i**) and 29 (**k**–**m**). Transverse section levels are at the fourth branchial arch (**d**–**f**), the second branchial arch (**g**–**i**) and the hyoid arch (**j**–**m**), as indicated in (**a**), (**b**) and (**c**). Arrowheads indicate HBM precursors. Pharyngeal arches and the pericardium were in the same transverse plane. Note that HBM precursors passed through the lateral side of pericardium, and then extended medially at the hyoid arch. Adp, anterodorsal lateral line placode; AER, apical ectodermal ridge; ba, branchial arch mesoderm; bbm, basibranchial mesenchyme; dm, dermomyotome; drg, dorsal root ganglion; eb, ectomesenchyme of branchial arches; ed., endolymphatic duct; eh, ectomesenchyme of hyoid arch; em, ectomesenchyme of mandibular arch; gb, gill bud; ha, hyoid arch mesoderm; he, heart; hg, hatching gland; ih interhyoideus; ma, mandibular arch mesoderm; ne, nasal epithelium; np, nasal prominence; nt, neural tube; ot, otic vesicle; pcc, pericardial cavity; pcm, pericardial mesoderm; pe, pharyngeal endoderm; pfm, pectoral fin muscle; pohm, postotic paraxial head mesoderm; scl, sclerotome; thy thyroid gland. Scale bars on whole embryos, 200 μm. Scale bars on sections, 50 μm

At stage 27, *S. torazame Pax3* marked HBM precursors that extended posterior to the pharyngeal arches and arrived at the lateral side of the pericardium. Section in situ hybridization clearly showed that the precursors did not intermix with the *Dlx5*-, *Hoxa2*- and *Tbx1*-positive branchial arches, and both pharyngeal arches and the pericardium were observed in the same transverse plane (Fig. 6a, d-f and Additional file 1: Figure S8a, b). The precursors passed through the pericardium laterally and reached to the second branchial arch level at stage 28 (Fig. 6b, g-I and Additional file 1: Figure S8c, d). By stage 29, *Pax3*-positive cells were found ventral to the pharynx and the anterior tip of the expression at the hyoid arch level showed weak staining (Fig. 6c). In section in situ hybridization, the *Pax3* expression was detected dorsomedial to the *Dlx5*-positive ectomesenchyme and *Tbx1* expressions localized in the interhyoideus (Fig. 6h–k). This indicates the medial extension of HBM precursors and the ventral covering of pharyngeal components at the hyoid arch level.

**Fig. 7:**
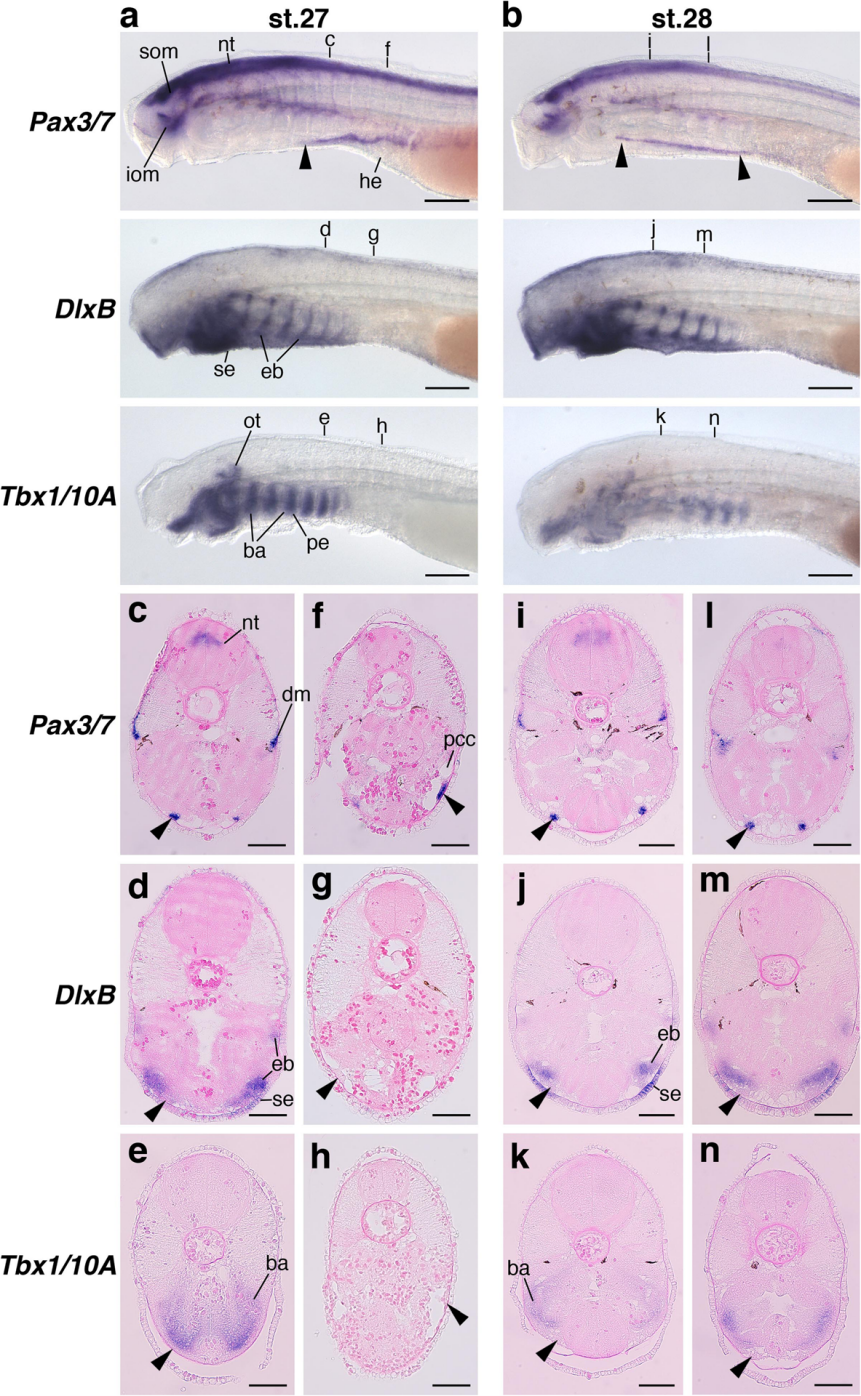
The arrangement of HBMs pathways and pharyngeal arches in lamprey embryos. *Pax3/7*, *DlxB* and *Tbx1/10A* expressions in lampreys at stage 27 (**a**) and 28 (**b**) from the lateral view. Transverse sections of stage 27 embryos at the fifth branchial arch (**c**–**e**) and heart (**f**–**h**) levels, and stage 28 embryos at the second (**i**-**k**) and fourth branchial arch (**ln**) levels. Section levels are indicated in (**a**) and (**b**). Arrowheads indicate HBM precursors. Pharyngeal arches and the pericardium were not in the same transverse plane, and HBM precursors expanded lateral to the pharyngeal arches. ba, branchial arch mesoderm; dm, dermomyotome; eb, ectomesenchyme of branchial arches; he, heart; iom, infraoptic muscle; nt, neural tube; ot, otic vesicle; pcc, pericardial cavity; pe, pharyngeal endoderm; se, surface ectoderm; som, supraoptic muscle. Scale bars on whole embryos, 200 μm. Scale bars on sections, 50 μm

The above observations are consistent with results from chicken and mouse embryos, in which developing HBMs circumvent the postotic pharyngeal arches and travel in the cephalic crest cell-free environment before reaching the hyoid arch. In contrast, lamprey *Pax3/7* expression labeled HBM precursors extending from the lateral side of the pericardium to the posterior pharynx (Fig. 7a–c, f, i, l). Lamprey *DlxB* and *Tbx1/10A* were detected in the pharyngeal arch ectomesenchyme and mesoderm, respectively (Fig. 7a, b). Analyses of histological sections after in situ hybridization revealed that *DlxB-*, *Hox2α-*, *Hox3α-* and *Tbx1/10A*–positive cells in the pharyngeal arches were observed dorsomedial to the *Pax3/7*-positive cells, and pharyngeal arches and the pericardium were not in the same transverse plane (Fig. 7c-n and Additional file 1: Figure S8e-n). Thus, the lamprey HBM precursors enter into the neural crest cell environment as soon as they pass through the pericardial domain located caudal to the entire pharynx.

Taken together, these results are consistent with our histological observations, indicating that shark HBM precursors pass outside of the pericardium and expand inside the pharynx at the level between the hyoid and first branchial arches, whereas in lamprey HBM precursors remain outside the pericardium and pharyngeal arches. Thus, although the initial avoidance of the branchial region and the lateral migration to the pericardium are likely to be steps in a conserved developmental program in the entire vertebrate HBMs [9, 24, 27, 30, 31], the trajectory of HBMs internalized at the hyoid level is a shared trait only for crown gnathostomes (Figs. 2, 3, 4, 6 and 7).

## Discussion

In this study, we show that HBM precursors travel along the head trunk interface, namely, along the posterior edge of the postotic pharyngeal arches and lateral to the pericardium in both crown gnathostomes and lampreys, while the topographical relationship of the pericardium to the pharynx and the trajectory of HBMs in the ventral pharynx differ substantially between the two groups. In the gnathostomes, the pericardium lies ventral to the pharyngeal arches, and the HBM precursors intrude inside the hyoid and mandibular arches (Figs. 2, 3, 4, 6). In contrast, in the lamprey, the pericardium is located caudal to the pharynx, and the HBM precursors extend outside of the pharyngeal muscles and skeletons (Figs. 2, 7). These observations provide unique insights into the evolution of the head–trunk interface as discussed below.

**Fig. 8:**
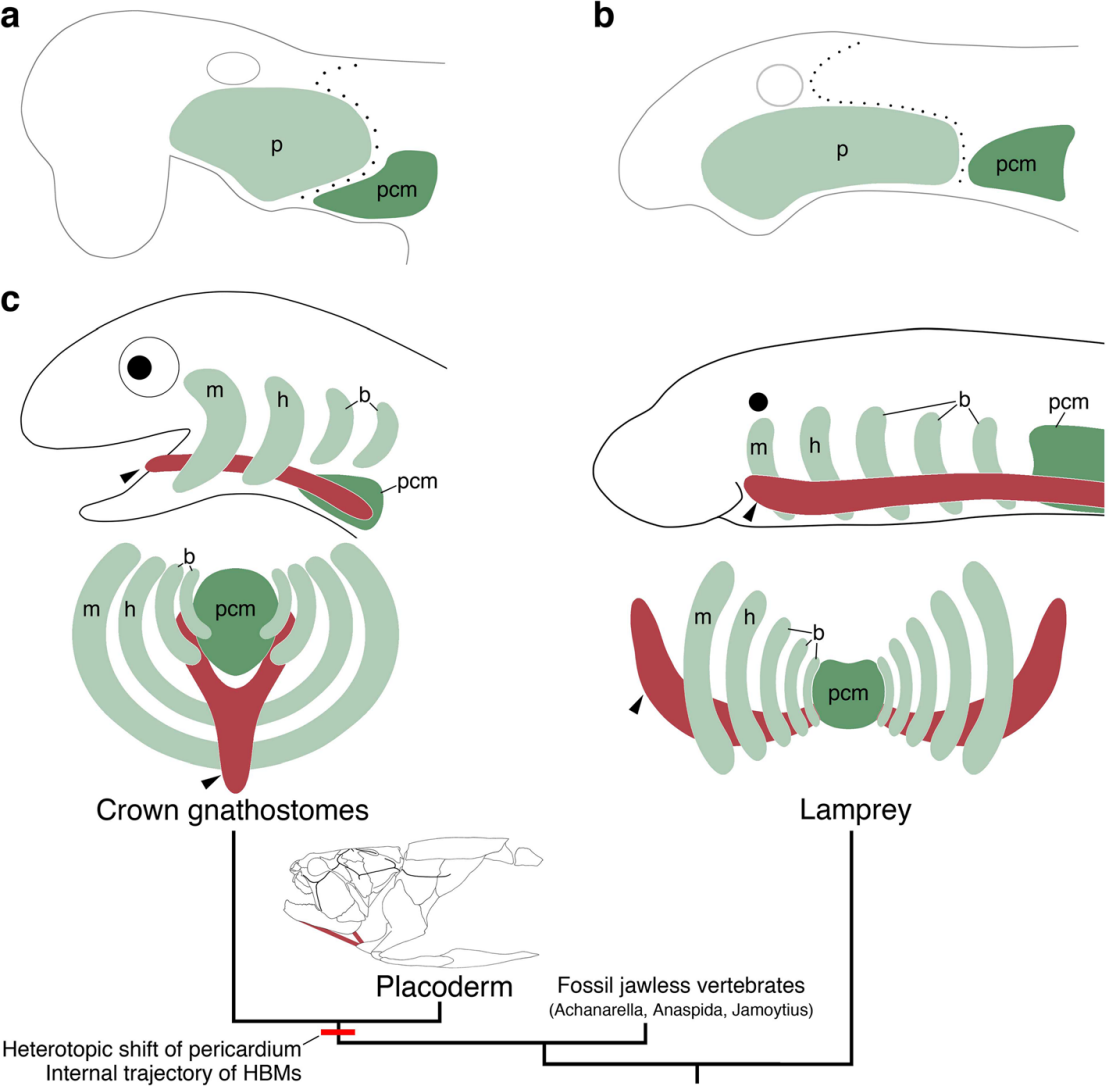
Evolution of the head–trunk interface and HBMs. Schematic representations of the vertebrate head–trunk interface (**a**, **b**). The pericardium is located ventral to the pharyngeal arches in crown gnathostomes (**a**), whereas the pericardium is posterior to the arches in the lamprey (**b**). Dotted lines indicate the head–trunk interface. **c** A phylogenetic tree (modified from [63, 68]) with lateral and anterior views of HBMs and pharyngeal arches in crown gnathostomes (left) and the lamprey (right). Arrowheads indicate HBMs. Putative HBMs are shown in red lines in placoderm [67]. b, branchial arch; h, hyoid arch; m, mandibular arch; p, pharyngeal arches; pcm, pericardial mesoderm

It has been reported that postotic pharyngeal arches adjoin occipital somites dorsally and the pericardium ventrally, creating the head–trunk interface in the vertebrate body (Fig. 1b) [1]. Our study reveals that the pericardial mesoderm is ventral to the pharyngeal domain in gnathostomes, but caudal to the pharynx in lamprey (Figs. 4, 5, Additional file 1: Figures S5 and S7). This spatial difference in the pericardium implies that the ventral part of the head–trunk interface, especially the boundary between the pharynx and pericardium, is distinct in gnathostomes and lamprey (Fig. 8a, b), suggesting that the head trunk boundary may not be constant in all vertebrates. In addition, the position of the pericardium is also involved in the development of HBMs. We found that, in all vertebrate embryos observed here, the HBM precursors definitely passed through the lateral side of the pericardium (Figs. 2, 3, 4, 6 and 7). The migration of HBM precursors lateral to the pericardium has also been observed in rays, salamanders, and mammals in previous histological analyses [9, 24, 27, 31, 33].

These findings indicate that the pericardium is tightly coupled with the development of HBMs in vertebrates. This linkage suggests that the position of the gnathostome pericardium may permit the HBM precursors to circumvent branchial arches ventrally and project medially into the hyoid arch to reach the inside of the mouth (Fig. 8). This trajectory is absent in the lamprey and inconceivable in the embryos given the position of the lamprey pericardium, which does not attach to the mandibular and hyoid arches (Figs. 4, 5, 7 and 8). Therefore, a heterotopic shift of pericardium relative to the pharynx may underlie a trajectory change of the HBMs.

The above discussion implies a morphological transition of the head–trunk interfacial domain from one state to another in vertebrate phylogeny. The prevalence of the pericardium, pharynx, and HBMs in extant vertebrates suggests the presence of those embryonic elements in a common ancestor of cyclostomes and gnathostomes, although the arrangement of ancestral elements remains unknown. In this regard, information from paleontology is important to speculate about the ancestral condition.

Some fossil jawless vertebrates, such as *Achanarella*, *Anaspida*, and *Jamoytius*, possess the posteriorly elongated branchial apparatus with a number of gill openings [60, 61], and trunk scales or myomeres dorsal to the branchial region [61–63], but lack apparent occipital and shoulder elements. These morphological features are shared by the interfacial domain of modern cyclostomes, although the hagfish likely represents a derived condition in the hypoglossal nerve and HBMs as well [1, 17].

It is difficult to speculate with confidence on the anatomy of the head–trunk interface and HBMs in other fossil jawless vertebrates, such as Heterostracans, Galeaspids and Osteostracans, because data on their soft tissue morphology are very rare, insights into their internatl anatomy must be gleaned mainly from endo- and exoskeletons [64]. Of these fossil species, Osteostracans have an ossified pericardium attached to the postbranchial wall, and the pericardium and posterior gills are positioned in the same longitudinal axis [60, 64]. They also possess the pectoral girdle, and possibly paired common cardinal veins (the ducts of Cuvier) just behind the postbranchial wall, suggesting a gnathostome-like condition of the head–trunk interface in this animal [54, 65, 66]. Thus, it may be conceivable that HBMs of Osteostracans are positioned on the ventral aspect of the pharynx, separated from the dorsal muscle mass, and caudally attached to the pectoral girdle, or possibly to the ventral bridge of dermal bone located ventral to the pericardium [64]. The morphology of anterior HBMs and their position relative to the gill elements are difficult to assume, and further analyses of this fossil will be required. In contrast, placoderms display muscle attachment sites for HBMs on the lower jaw and shoulder skeletons, which are comparable with those of extant gnathostomes [67].

Given the current phylogenetic positions of the abovementioned fossil vertebrates [63, 68], these observations suggest that the head–trunk interface found in the lamprey embryo may represent the ancestral condition, and a rearrangement of preexisting embryonic components in the ancestral vertebrate may have occurred during the transition from jawless to jawed vertebrates (Fig. 8c). Thus, we hypothesize that a heterotopic shift of the pericardium together with a gain of an internal trajectory of HBM precursors, was a key embryonic change for the evolution of the gnathostome HBMs, which function as jaw opening and tongue muscles (Fig. 8c) [7, 11, 13, 14]. Furthermore, this rearrangement may have contributed to the establishment of the gnathostome head–trunk interface, which provides the basis for the development of the jawed vertebrate neck.

## Conclusions

In our comparative embryological analyses, we found that the pericardium was posterior to the pharynx and HBM precursors extended outside of those elements in the lamprey, whereas the pericardium was ventral to the pharynx and HBM precursors traveled outside of the pericardium and inside the hyoid and mandibular arches in crown gnathostomes. Based on current paleontological data on stem gnathostomes, we hypothesize that the embryonic arrangement of the pericardium, pharynx, and HBM precursors likely underwent a transition from the lamprey condition to the crown gnathostome condition, which was a key embryonic change not only for the establishment of the head–trunk interface, but also for the evolution of the neck in jawed vertebrates.

## Additional file

Additional file 1: Supplementary materials. (PDF 6266 kb)
