# Supplementary material for: Development of hypobranchial muscles with special reference to the evolution of the vertebrate neck
Source: Zoological Lett. 2018 Feb 18;4:5. doi: 10.1186/s40851-018-0087-x (PMC5816939; doi:10.1186/s40851-018-0087-x)
Supplement: Supplementary file 1 — Supplementary materials. (PDF 6266 kb) [file 40851_2018_87_MOESM1_ESM.pdf]

Additional file 1

Development of hypobranchial muscles with special reference to the evolution of the vertebrate neck

Noritaka Adachi, Juan Pascual-Anaya, Tamami Hirai, Shinnosuke Higuchi, and Shigeru Kuratani

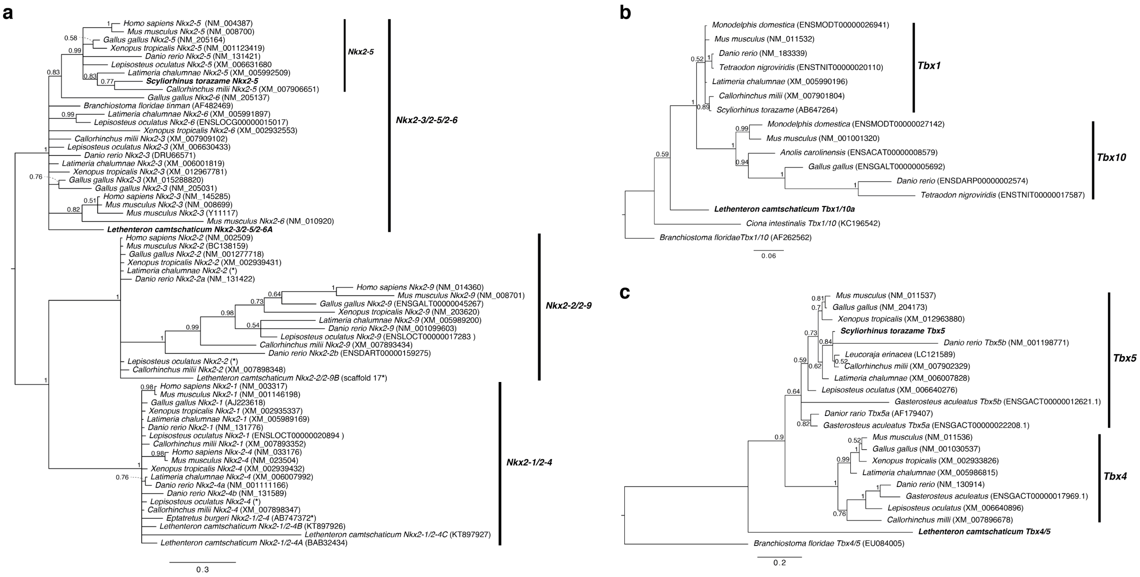

Figure S1. Bayesian inference phylogenetic trees of lamprey and shark genes. a, A 10 million generation tree (7,500,000 million post-burn trees) of NKX2-3, NKX2-5, NKX2-6, NKX2-1, NKX2-2, NKX2-4, NKX2-9 protein sequences. The tree was rooted on the ingroup consisting on ((NKX2-1, NKX2-4)(NKX2-2, NKX2-9)). While *S. torazame* gene is clearly a *Nkx2-5* orthologue, the lamprey counterpart is of uncertain orthology, so we have named it *Nkx2-3/2-5/2-6A*. **b**, A 1.5 million generation tree (1,125,000 post-burn trees) of TBX1 and TBX10 protein sequences. TBX1/10 from the amphioxus *B. floridae* and the ascidian *Ciona intesinallis* were included as vertebrate outgroup sequences. **c**, A 2 million generation tree (1,500,000 post-burn trees) of TBX4 and TBX5 protein sequences. TBX4/5 from the amphioxus *B. floridae* was included as an outgroup. In bold, sequences described in this study; accession numbers of known sequences used in these phylogenetic analyses are included inside parentheses next to each branch. Asterisk indicates sequences predicted using genomic sequences in public databases.

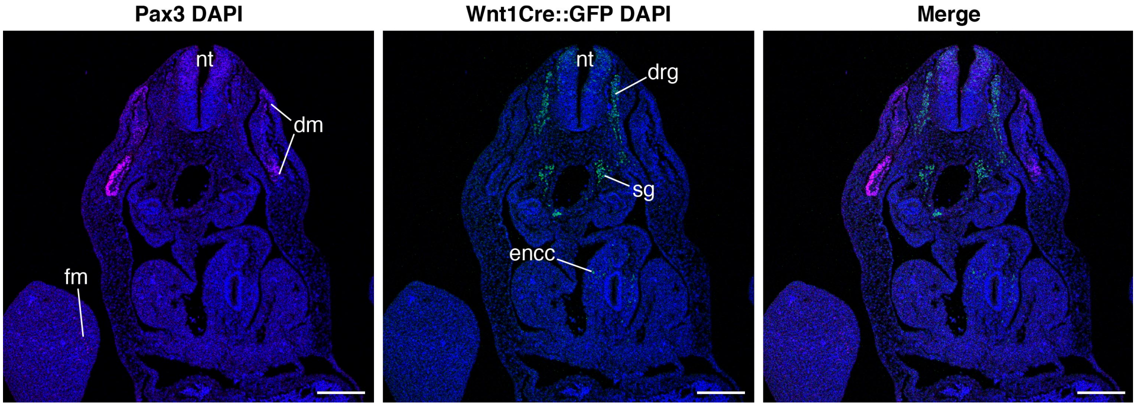

Figure S2. Transverse sections at the interlimb level of E10.5 Wnt1-Cre/R26R-H2B-EGFP mouse embryos. PAX3 protein was detected in the dorsal part of the neural tube, dermomyotome, and forelimb muscles, and EGFP protein was observed in the roof plate, dorsal root ganglion, sympathetic ganglion, and enteric neural crest cells. dm, dermomyotome; drg, dorsal root ganglion; encc, enteric neural crest cells; fm, forelimb muscles; nt, neural tube; sg, sympathetic ganglion. Scale bars, 200 µm.

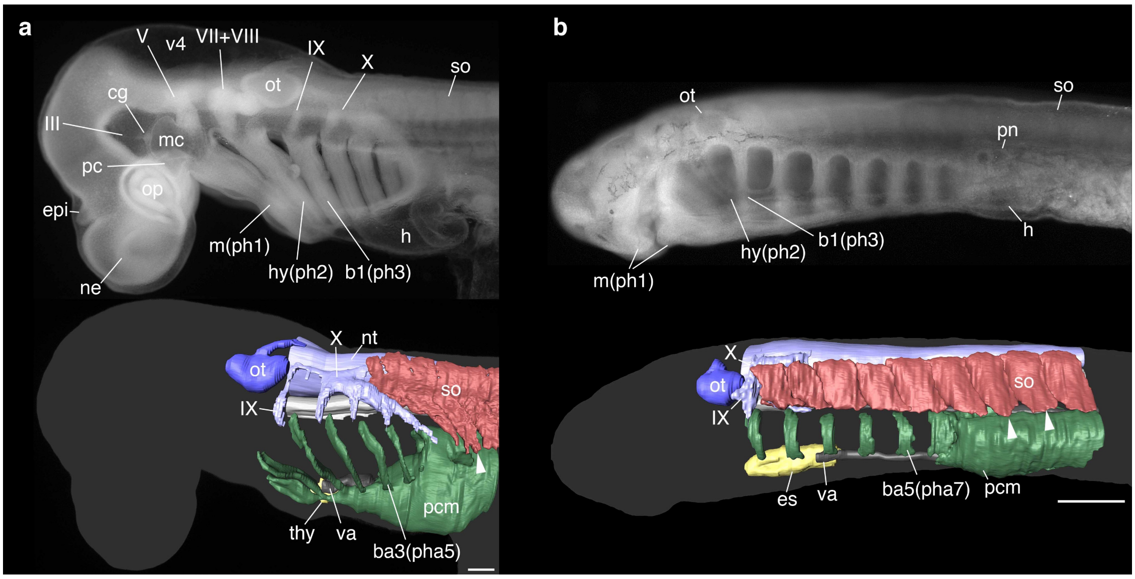

Figure S3. 3D reconstruction of shark and lamprey embryos. The lateral view of embryos with 3D reconstruction images from otic to heart level in the shark (**a**) and the lamprey (**b**). Arrowheads indicate HBMs. In this study, we use terminology of pharynx as follows: the mandibular arch is the first pharyngeal arch, the hyoid arch is the second pharyngeal arch, and the first branchial arch is the third pharyngeal arch. b1, first branchial arch; ba3-5, third to fifth branchial arch mesoderm; cg, ciliary ganglion; epi, epiphysis; es, endostyle; h, heart; hy, hyoid arch; m, mandibular arch; mc, mandibular head cavity; ne, nasal epithelium; nt, neural tube; op, optic vesicle; ot, otic vesicle; pc, premandibular head cavity; pcm, pericardial mesoderm; ph1-3, first to third pharyngeal arch; pha5-7, fifth to seventh pharyngeal arch mesoderm; pn, pronephros; so, somite; thy, thyroid gland; va, ventral aorta; v4, fourth ventricle; III, oculomotor nerve; V, trigeminal nerve; VII+VIII, facial and vestibulocochlear nerve; IX, glossopharyngeal nerve; X, vagus nerve. Scale bars, 200 µm.

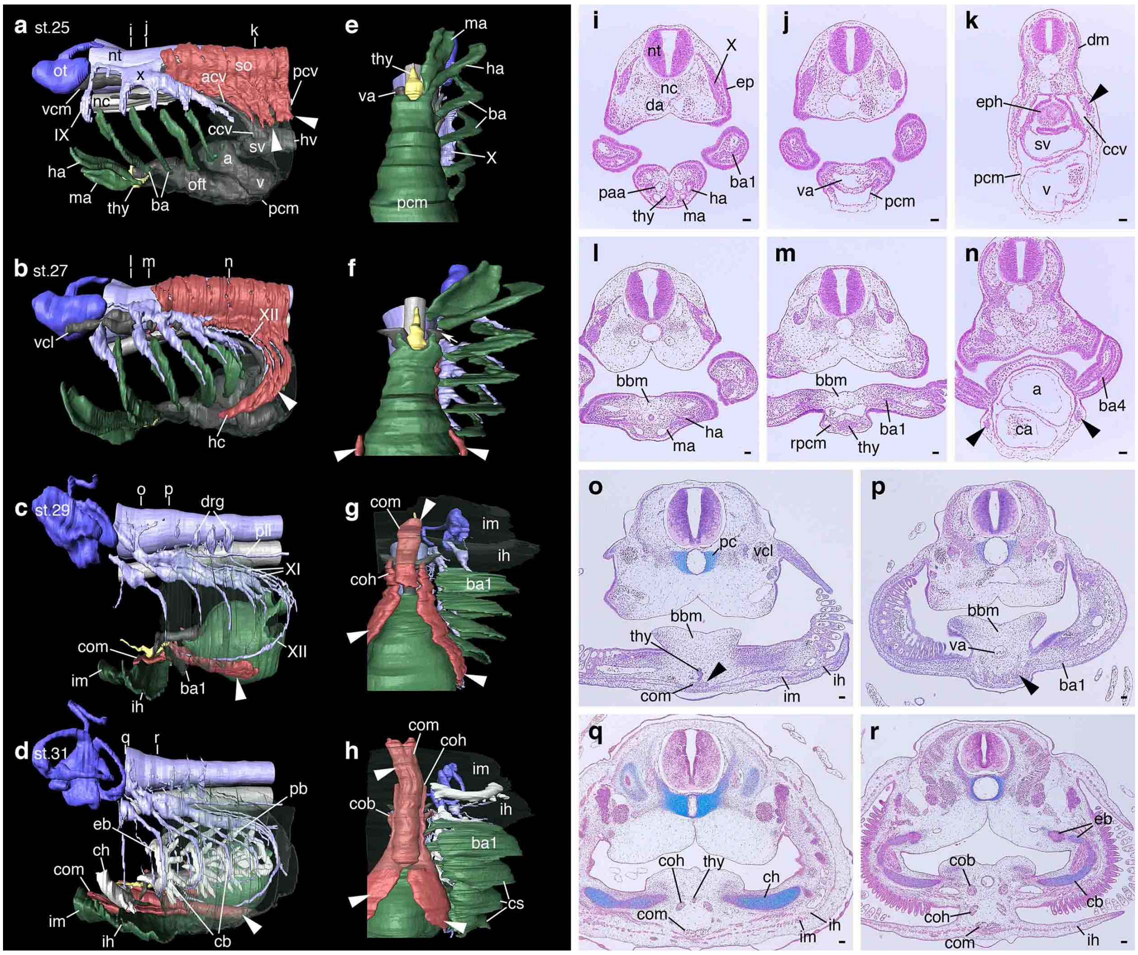

Figure S4. Morphological and histological analysis of shark embryo. 3D reconstruction images of developing shark embryos from lateral view (**a**-**d**) and ventral view (**e**-**f**). The pericardial mesoderm (**a**, **b**), branchial arch mesoderm (**c**, **d**), and ventral gill muscles (**g**, **h**) are translucent. Transverse sections (**i**-**r**) taken at the level indicated in (**a**-**d**). Arrowheads indicate HBMs. a, atrium; acv, anterior cardinal vein; ba, branchial arch mesoderm; ba1, first branchial arch mesoderm; ba4, fourth branchial arch mesoderm; bbm, basibranchial mesenchyme; cb, ceratobranchial; ccv, common cardinal vein; ch, ceratohyal; cob, coracobranchialis; coh, coracohyoideus; com, coracomandibularis; cs, constrictor superficialis; da, dorsal aorta; dm, dermomyotome; drg, dorsal root ganglion; eb, epibranchial; ep, epibranchial placode; eph, esophagus; ha, hyoid arch mesoderm; hc, hypoglossal cord; hv, hepatic vein; ih interhyoideus; im, intermandibularis; ma, mandibular arch mesoderm; nt, neural tube; nc, notochord; oft, outflow tract; ot, otic vesicle; pb, pharyngobranchial; pcm, pericardial mesoderm; pcv, posterior cardinal vein; pll, posterior lateral line; rpcm, remnants of pericardial mesoderm; so, somite; sv, sinus venosus; thy, thyroid gland; v, ventricle; va, ventral aorta; vcl, vena capitis lateralis; vcm, vena capitis medialis; IX, glossopharyngeal nerve; X, vagus nerve; XI, accessory nerve; XII, hypoglossal nerve. Scale bars, 50 µm.

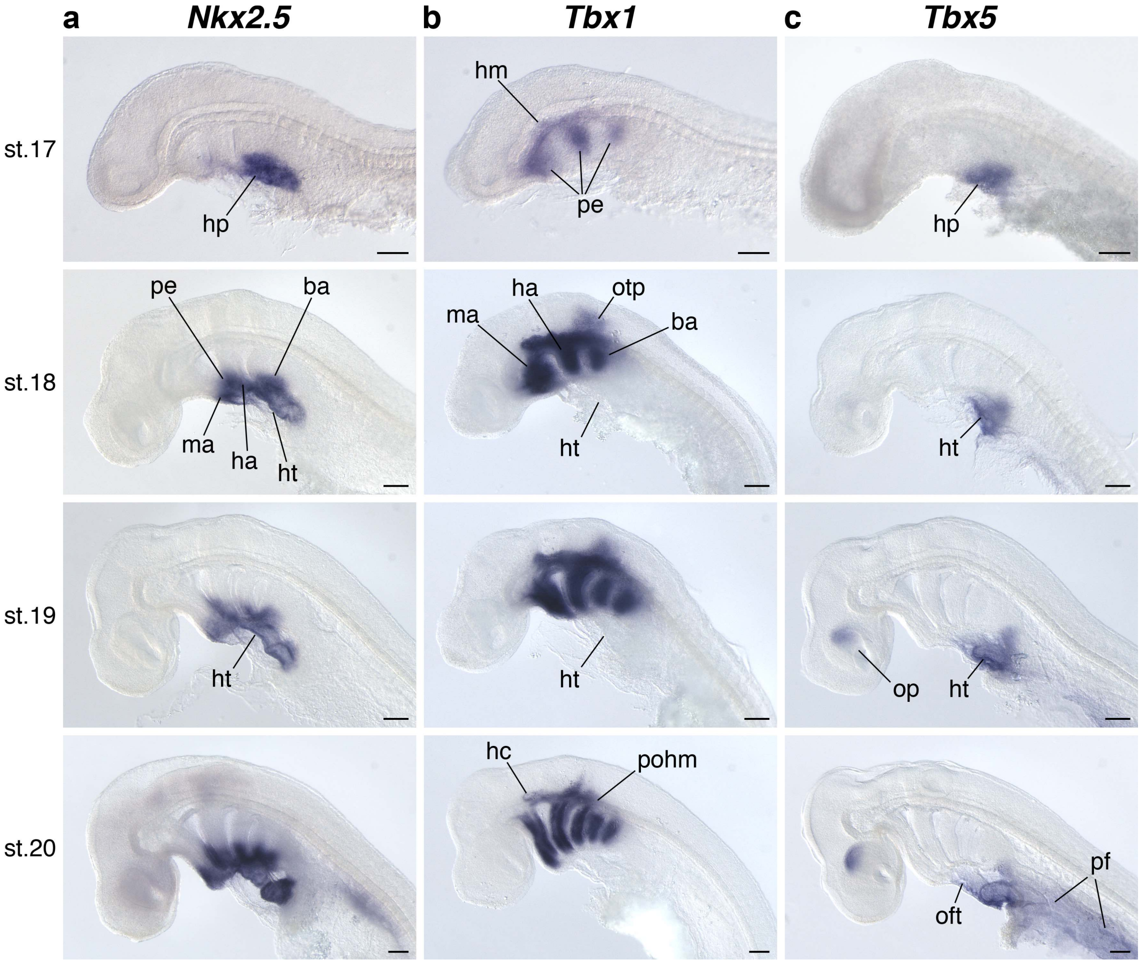

Figure S5. The relative position of the pericardium and pharyngeal arches in shark embryo. Gene expression patterns of shark *Nkx2-5* (**a**), *Tbx1* (**b**) and *Tbx5* (**c**) at stages 17 to 20. *Nkx2-5* expression was found in the heart primordium, heart tube, pharyngeal endoderm, and pharyngeal arch mesoderm (**a**). The rostral part of heart tube was close to the mandibular and hyoid arch mesoderm. *Tbx1* was expressed in the hyoid head mesoderm, otic placode/vesicle, pharyngeal endoderm, and pharyngeal arch mesoderm (**b**). *Tbx5* was expressed in the heart primordium, heart tube, pericardial mesoderm, dorsal part of eye, and pectoral fin field (**c**). *Tbx5* expression was tapered at the outflow tract. *Tbx5* heart expression was found ventral to *Tbx1* expression domain and the rostral part of heart tube was in the vicinity of mandibular and hyoid arch mesoderm. ba, branchial arch mesoderm; ha, hyoid arch mesoderm; hc, hyoid head cavity; hm, hyoid head mesoderm; hp, heart primordium; ht, heart tube; ma, mandibular arch mesoderm; oft, outflow tract; op, optic vesicle; otp, otic placode; otv, otic vesicle; pcm, pericardial mesoderm; pe, pharyngeal endoderm; pf, pectoral fin field; pohm, postotic paraxial head mesoderm. Scale bars, 200 μm.

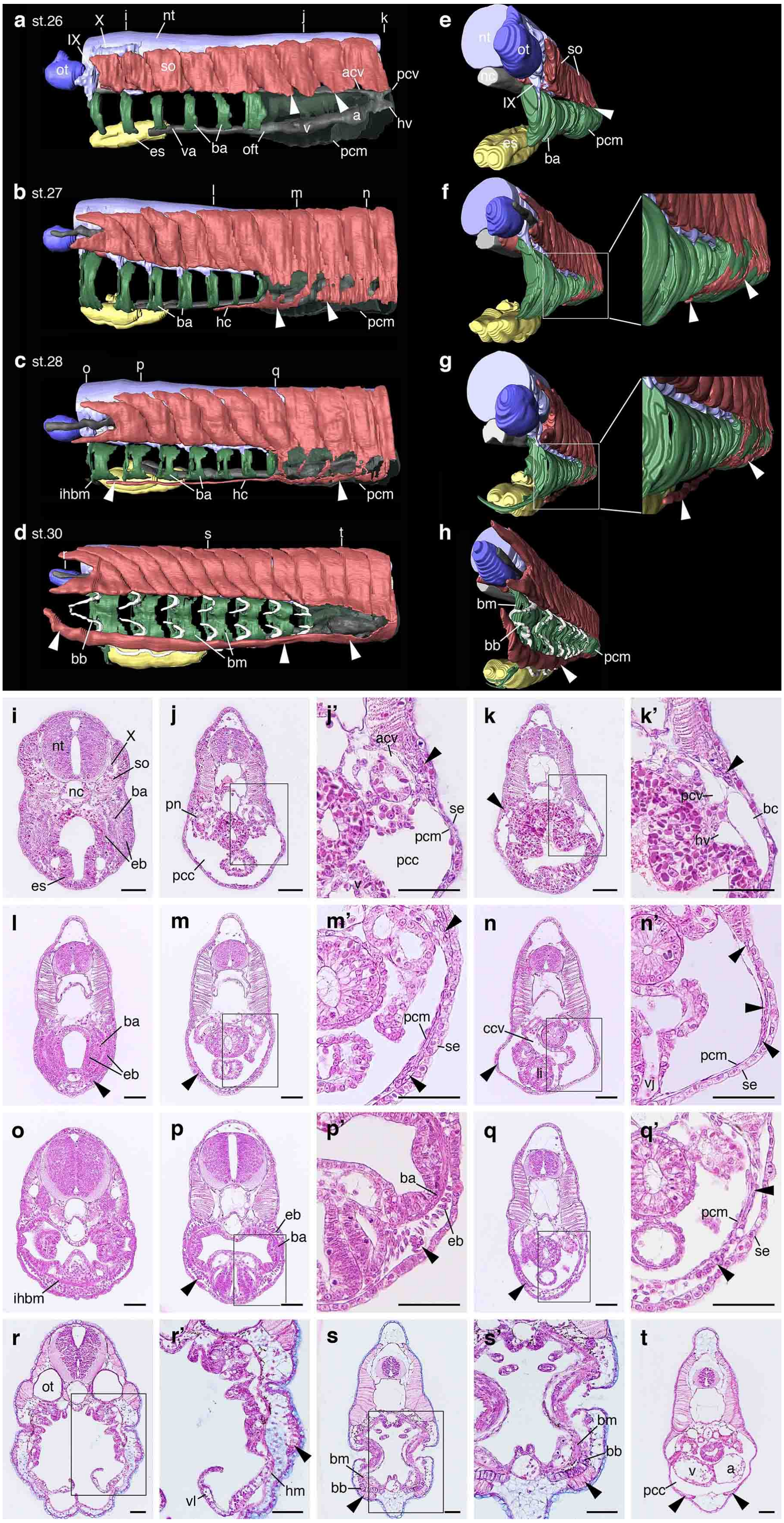
a st.26
b st.27
c st.28
d st.30
e
f
g
h
nt
ot
so
IX
X
acv
pcv
hv
a
v
va
ba
oft
pcm
es
hc
ihbm
bb
bm
nc
eb
pn
pcc
se
bc
ccv
li
vj
hm
vl
i
j
j'
k
k'
l
m
m'
n
n'
o
p
p'
q
q'
r
r'
s
s'
t

Figure S6. Morphological and histological analysis of lamprey embryo. 3D reconstruction images of lamprey embryos from lateral view (**a**-**d**) with translucent pericardial mesoderm, and anterior oblique view (**e**-**h**) with magnification of HBMs (**f**, **g**). Transverse sections (**i**-**t**) taken at the level indicated in (**a**-**d**). The higher magnification (**j'**-**s'**) of box in (**j**-**s**). Arrowheads indicate HBMs, which pass thought the outside of pericardial mesoderm and pharyngeal arches. Unlike previous observations and assumptions, the lateral plate mesoderm of lamprey shows the splanchnopleure and body cavity just caudal to the heart (**k**, **k'**), where jawed vertebrates possess the pectoral fin. a, atrium; acv, anterior cardinal vein; ba, branchial arch mesoderm; bb, branchial basket; bc, body cavity; bm, branchial muscles; ccv, common cardinal vein; eb, ectomesenchyme of branchial arches; es, endostyle; hc, hypoglossal cord; hm, hyoid arch muscle; hv, hepatic vein; ihbm, interhyobranchial mesoderm; li, liver; nt, neural tube; nc, notochord; ot, otic vesicle; pcc, pericardial cavity; pcm, pericardial mesoderm; pcv, posterior cardinal vein; pn, pronephros; se, surface ectoderm; so, somite; v, ventricle; va, ventral aorta; vl, velum; vj, ventral jugular vein; IX, glossopharyngeal nerve; X, vagus nerve. Scale bars, 50 μm.

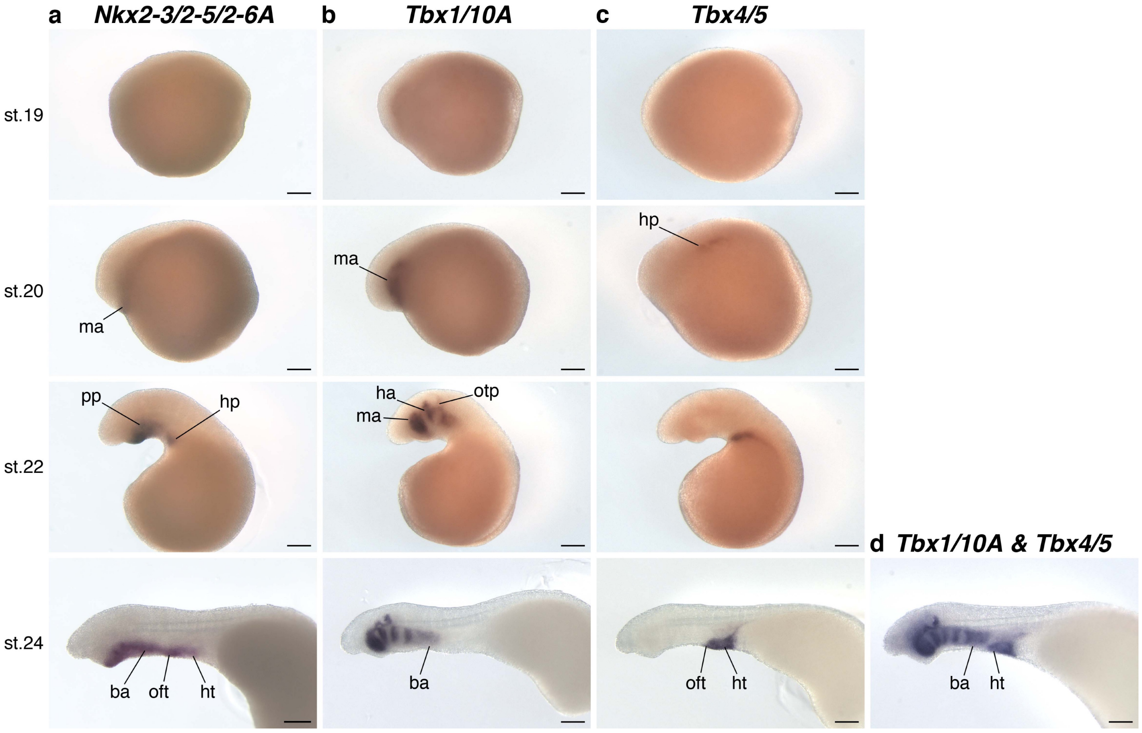

Figure S7. The relative position of the pericardium and pharyngeal arches in lamprey embryo. Gene expression patterns of lamprey *Nkx2-3/2-5/2-6A* (**a**), *Tbx1/10A* (**b**, **d**) and *Tbx4/5* (**c**, **d**) at stages 19 to 24. mRNA of *Nkx2-3/2-5/2-6A* was detected in the ventral part of pharyngeal arch mesoderm, pharyngeal pouches, and heart mesoderm (**a**). *Tbx1/10A* was expressed in the otic placode/vesicle and pharyngeal arch mesoderm (**b**), and *Tbx4/5* transcripts were detected in the heart primordium and heart tube (**c**). *Tbx4/5* signals were situated posterior to *Tbx1/10A* expression domain (**b**, **c**), and the rostral part of heart tube was located adjacent to the caudalmost branchial arch mesoderm (**a**, **d**). ba, branchial arch mesoderm; ha, hyoid arch mesoderm; hp, heart primordium; ht, heart tube; ma, mandibular arch mesoderm; oft, outflow tract; otp, otic placode; otv, otic vesicle; pp, pharyngeal pouch. Scale bars, 200 μm.

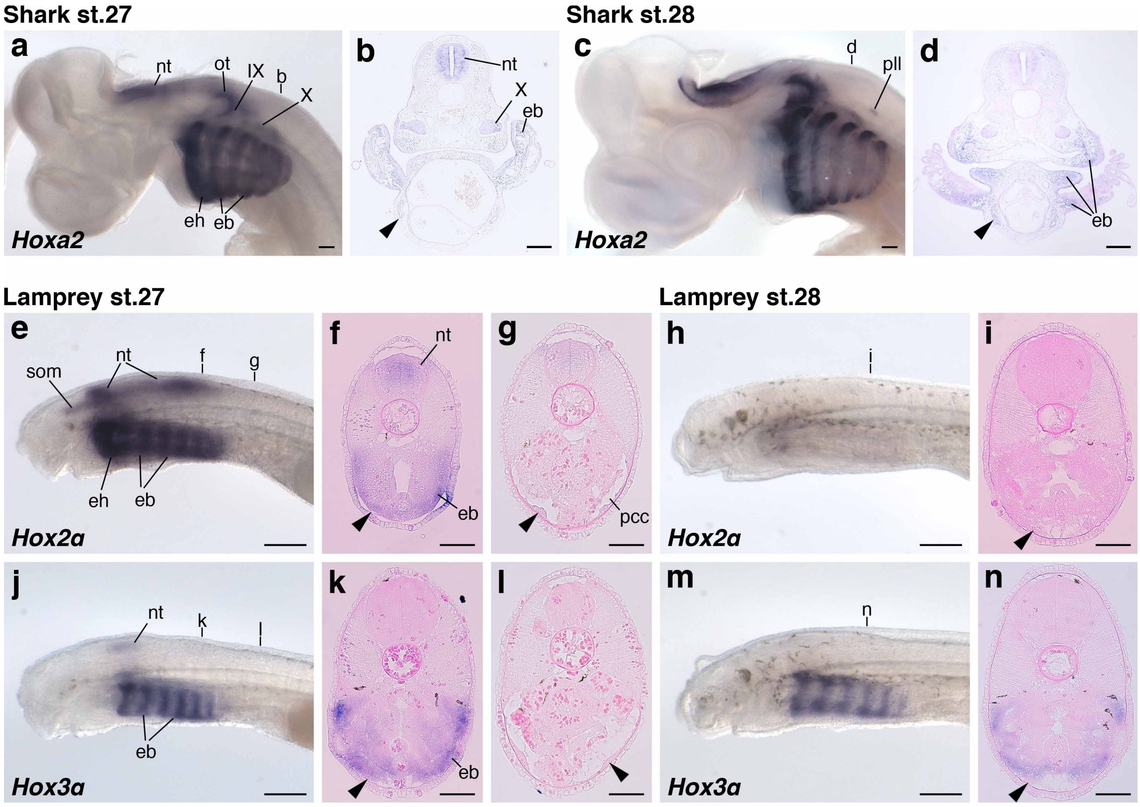

Figure S8. The arrangement of HBMs pathways and pharyngeal arches in shark and lamprey embryos. Expression pattern of *Hox* genes in shark (**a**-**d**) and lamprey (**e**-**n**) embryos. *S. torazame Hoxa2* was detected in the neural tube, otic vesicle, glossopharyngeal nerve, vagus nerve, ectomesenchyme of hyoid and branchial arches, and posterior lateral line (**a**-**d**). Lamprey *Hox2α* was expressed in the supraoptic muscle, neural tube, and ectomesenchyme of hyoid and branchial arches at the stage 27 (**e**-**g**), and slightly detected in the ectomesenchyme of hyoid arch at the stage 28 (**h**, **i**). Lamprey *Hox3α* expression was found in the neural tube and ectomesenchyme of branchial arches (**j**-**n**). eb, ectomesenchyme of branchial arches; eh, ectomesenchyme of hyoid arch; nt, neural tube; ot, otic vesicle; pcc, pericardial cavity; pll, posterior lateral line; som, supraoptic muscle; IX, glossopharyngeal nerve; X, vagus nerve. Scale bars on whole embryos, 200 µm. Scale bars on sections, 50 µm.

Gene	Forward primer	Reverse primer
L. camtschaticum		
Hox2alpha	GTCTCACCTCGTCCTCCTCT	GGTCCAGCGTGCTCTCTAAG
Nkx2-3/2-5/2-6A	AGACATCCTCAACCTCGAACATCAGCAACATCACC	ACACCTGCGCCTGCGAGAAGAGCACC
Pax3/7	GTGTTTATGCAGCAGAGTTCTCAGGATG	TTGGCCAAGTAGTCAGCGGCAGTCTGTC
Tbx1/10A	GTCCAAGCAACATCGTCAACATCATCATCATCG	GACGCGACATTAGGGGCAGGTGAGC
Tbx4/5	CAGACAACAAGTGGAGTGTGAGCGG	CTTGGCACTGCTCGTCATCCTGGAA
S. torazame		
Hoxa2	ACTCGACGCTTTCACACTCGACAGT	GAAGGCATATTCAGCTCACACCGACTT
Nkx2-5	GCTGAGGTCACTTTGGATGCAGTAGTTGG	CGTTCTGATTGTATTGCCGAGCTGTCC
Tbx5	CCTTGGGGTTTTGGTGCCTCTTTTGC	CTGGCGAGAAACCGAGCTCTGATGC
M. musculus		
Myf5	CCCAGGAATATATAAAGAGCCCCAACCTC	TCAGTTAAATCTATTTTCTCATAAAGTGGCAAGAC
Pax3	TCGTCTCGCCTTCACCTGGATATAATTTGC	ACATGCCTCCAGTTCCCCGTTCTCAAGC
G. gallus		
Myf5	GTGATGGACAGCTGCCAGTTCTCCC	GCCACTCTGCTCCGTCGCGTAG
Pax3	GCTGCCCAACCATATCCGCCACAAGATCG	GTGGTGCTATAGGTCGGTGGGCAGTAGG

Gene	Accetion number
L. camtschaticum	
Nkx2-3/2-5/2-6A	LC333760
Tbx1/10A	LC331563
Tbx4/5	LC331564
S. torazame	
Hoxa2	MF398238
Nkx2-5	LC331565
Tbx5	LC331566

Supplemental table.
